# Development of a Screening Method for Health Hazard Ranking and Scoring of Chemicals Using the Mahalanobis–Taguchi System

**DOI:** 10.3390/ijerph15102208

**Published:** 2018-10-10

**Authors:** Da-An Huh, Hong Lyuer Lim, Jong-Ryeul Sohn, Sang-Hoon Byeon, Soonyoung Jung, Woo-Kyun Lee, Kyong Whan Moon

**Affiliations:** 1 Department of Health Science, Korea University, Anam-ro 145, Seongbuk-gu, Seoul 02841, Korea; black1388@korea.ac.kr (D.-A.H.); limhl1213@naver.com (H.L.L.); sohn1956@korea.ac.kr (J.-R.S.); shbyeon@korea.ac.kr (S.-H.B.); 2 Department of Computer Science and Engineering, Korea University, Anam-ro 145, Seongbuk-gu, Seoul 02841, Korea; jsy@korea.ac.kr; 3 Department of Environmental Science and Ecological Engineering, Korea University, Anam-ro 145, Seongbuk-gu, Seoul 02841, Korea; leewk@korea.ac.kr

**Keywords:** chemical ranking and scoring, Mahalanobis–Taguchi System, priority setting

## Abstract

For efficient management of chemicals, it is necessary to preferentially select hazardous chemicals as being high-priority through a screening method. Over the past 20 years, chemical ranking and scoring (CRS) methods have been applied in many countries; however, these CRS methods have a few limitations. Most of the existing methods only use some of the variables to calculate the hazard of chemicals or use the most conservative score without consideration of the correlation between chemical toxicities. This evaluation could underestimate or overestimate the real health hazard of the chemicals. To overcome the limitations of these methods, we developed a new CRS method using the Mahalanobis–Taguchi System (MTS). The MTS, which conducts multivariate analysis, produced chemical rankings that took into accounts the correlation between variables related to chemical health hazards. Also, the proportion of chemicals managed by the Korea Chemicals Control Act that were given a high rating appeared to be higher when the MTS was used, compared to the existing methods. These results indicated that the new method evaluated the health hazards of chemicals more accurately, and we expect that the MTS method could be applied to a greater range of chemicals than the existing CRS methods.

## 1. Introduction

The number of hazardous chemicals has been steadily increasing with the production of new chemicals. About 200 to 300 new chemicals are produced each year, and about 45,000 types of chemicals are used in Korean workplaces [1]. The chemicals should be controlled systemically because of their diverse hazards, but it takes a large amount of time and resources to evaluate the hazard of chemicals individually. Therefore, for efficient management of chemicals, it is necessary to preferentially select hazardous chemicals as being high-priority through a screening method [2,3,4,5]. Over the past 20 years, chemical ranking and scoring (CRS) methods have been applied in many countries [6,7,8,9,10,11]. The European Union proposed the European Union Risk Ranking Method (EURAM) [12], and the Environmental Protection Agency introduced the Chemical Hazard Evaluation for Management Strategies (CHEMS) [4] for selecting high-priority chemicals. In Korea, the Korean Chemical Ranking and Scoring System has been developed and applied in the field [8].

However, these CRS methods have some limitations. There are many variables related to the health hazard of chemicals, but most of these methods only use some of the variables to calculate the health hazard of chemicals. This evaluation could underestimate the real health hazard of the chemicals. Another limitation is that the methods use the most conservative value in scoring or do not consider the correlation with toxicity. For example, the EURAM only includes carcinogenicity, which is more conservative than acute toxicity on the health hazard score, even if a chemical has both acute toxicity and carcinogenicity [12]. This evaluation could underestimate the actual health hazard of chemicals because information on acute toxicity is not included. Similarly, the CHEMS individually applies oral acute toxicity and inhalation acute toxicity in health hazard scores [4]. However, this could overestimate the real hazard because the two values have a positive correlation [13].

This study aimed to present a new CRS method that can evaluate the health hazard of chemicals comprehensively using the Mahalanobis–Taguchi System (MTS), which conducts multivariate analysis. This statistical approach can simultaneously use diverse information related to the health hazard of chemicals and calculate the hazard ranking with consideration of the correlations between variables. In this paper, we present the calculated health hazard rankings of chemicals using the current method, a comparison of this result with the methods of previous studies, and an interpretation of the meaning of the results. We expect that this new method could be applied to a greater range of chemicals compared with the existing methods, and that more exact priorities could be evaluated.

## 2. Materials and Methods

### 2.1. Introduction to the Mahalanobis–Tagichi System

The MTS was introduced by Genichi Taguchi. The MTS is a diagnosis and forecasting technique that uses multivariate data [14,15]. This method results in a Mahalanobis distance (MD) scale used to measure the level of abnormality of abnormal items compared with a group of normal items. The first step to calculate MDs is constructing the Mahalanobis space (MS), which is used as a reference group and should be similar and have common characteristics. Then, MDs can be calculated using the distance between the center of the MS and each item that is being evaluated. If an item has a small MD, then it can be said that the item has similar characteristics to the normal items. On the other hand, if another item has a large MD, then the item could have different characteristics compared with the normal items.

The MTS method is appropriate for evaluating the priorities of the health hazards of chemicals because of the following two aspects: First, the health hazards of chemicals should be estimated comprehensively using a variety of information, such as acute toxicity, carcinogenicity, mutagenicity, and reproductive toxicity. MD, the core concept of the MTS method, is the value that summarizes the multidimensional characteristics of the health hazard of chemicals into one simple value. The advantage of the MD is that it considers correlations between the variables, which are essential in pattern analysis [16,17,18,19]. Second, chemical hazards should be evaluated relatively because there is no absolute standard for determining chemical hazards. The MD is calculated by the distance from the center of the MS. Therefore, if a MS is constructed with chemicals that are relatively less harmful, then the larger the MD, the more harmful the chemical.

The MTS can be extended by combining the MD with Taguchi’s orthogonal array; this array is used to reduce the number of variables in multivariate systems. This study was concerned only with the process of calculating the ranking of chemical health hazards using the MD.

### 2.2. Data Sources and Chemical Variables

The health hazards of chemicals were classified according to the Globally Harmonized System (GHS). The GHS classifies chemical health hazards into 15 variables. Each variable has different classification criteria, and Category 1 indicates the highest hazard. If the hazard of a chemical is outside the classification criteria, then it is classified as Not Classified. If a chemical does not have sufficient hazard information, then it is classified as Classification Not Possible, and if a chemical is outside the definition of the GHS, then it is classified as Not Applicable [20].

In this study, we used 3028 out of 3967 chemicals provided by the National Institute of Technology and Evaluation after excluding those that did not have a CAS number and those that had overlapping chemicals. We excluded 3 (respiratory sensitization, skin sensitization, and aspiration hazard) of the 15 variables for health hazards because for these variables, more than 75% of the chemicals were classified as Classification Not Possible. In the case of inhalation acute toxicity, we incorporated three variables (gas, vapor, and dust and mist) into the most conservative value and named it acute toxicity (inhalation). Finally, we used 10 variables, as shown in Table 1, to calculate the overall health hazard of the chemicals.

### 2.3. Quantification of Health Hazard Variables

We quantified the GHS classification to calculate the MD. Regardless of the variables, Category 1, the most hazardous classification, was scored as 100 points, and the smaller the hazard, the smaller the scores that were allocated. Not Applicable was assigned the lowest score of 1 point, and Not Classified was assigned 10 points, which was lower than Category 5. In the case of Classification Not Possible, we assigned 30 points, which was close to the value of Category 3 because the absence of information does not mean that there is no hazard. Table 2 shows the results of the quantification of health hazards.

### 2.4. Construction of the Mahalanobis Space

The first step to calculate MDs is constructing the unit space, called the MS, which is used as a reference group. The unit space should be established using chemicals that have a relatively low health impact because all chemicals pose some health hazards.

In this study, the following criteria were applied to select chemicals with relatively low health hazards. First, we excluded substances that had one or more Category 1 or Category 2 classifications, which means they pose a high hazard. Second, the chemicals with a total score of 280 or less for the 10 health hazard variables were selected. For example, flutolanil (CAS No. 66332-96-5) had nine Not Classified (10 points) rankings and one Classification Not Possible (30 points) ranking. Therefore, the total score of flutolanil was 120, and we selected this chemical as the normal group. According to these criteria, we selected 151 chemicals as the normal group. Table 3 shows the process of selecting chemicals that are relatively less hazardous as the normal group.

### 2.5. Comparison of the Results Using Other Chemical Ranking and Scoring Methods

To verify the reliability of the MTS, we assessed the agreement between the results of the MTS and existing methods. The existing methods that we used for comparison were the EURAM and CHEMS. The EURAM separates the hazard score for the environment and human health, and the priority setting is conducted separately for each subject. Since the chemical information used in this study used information only related to health hazards, the EURAM also produced only a human health ranking for chemicals. On the other hand, the CHEMS calculates the total hazard score by combining the human toxicity score and the environmental toxicity score; that is, it is not possible to use only the human toxicity score for priority setting. Therefore, for the CHEMS, we evaluated the chemical hazard ranking by using all the information on environmental and human health.

There were four aspects that we wanted to compare between the MTS and the existing methods. First, we identified the agreement between hazard priorities for chemicals using the MTS, EURAM, and CHEMS. Second, we divided the chemical rankings into four classes according to the classification methods. Then, we checked the agreement of the chemical classes calculated by the CRS methods. The classification methods that we used were equal interval classification, quartile classification, Jenks natural breaks classification, and geometrical interval classification. Third, we classified the chemicals into five categories according to the Korea Chemicals Control Act and compared the average MD, EURAM hazard score, and CHEMS hazard score. The classification according to the Korea Chemicals Control Act was as follows: (i) chemicals requiring preparation for accidents (chemicals highly likely to cause chemical accidents due to their high acute hazard, explosiveness, etc. or likely to cause severe damage where a chemical accident occurs); (ii) toxic chemicals (hazardous chemicals prescribed and publicly notified by the Minister of Environment); (iii) prohibited chemicals (chemicals recognized as having a high risk if they are used for specific purposes, which are designated and publicly notified by the Minister of Environment to prohibit the manufacture, importation, sale, keeping, storage, transport, or use of such chemicals for all purposes); (iv) restricted chemicals (chemicals recognized as posing a high risk if they are used for specific purposes, which are designated and publicly notified by the Minister of Environment to prohibit the manufacture, importation, sale, keeping, storage, transport, or use of such chemicals for such purposes) [21]; and (v) other chemicals (chemicals not regulated by the Korea Chemicals Control Act). Finally, we compared the number of chemicals that were selected as being highly hazardous in each method that were also included in the chemical list of the Korea Chemicals Control Act.

### 2.6. Selection of Chemicals for Comparison

To compare the ranking of chemicals using the MTS, EURAM, and CHEMS, we extracted the common chemicals that could be calculated as hazard priorities using all three methods. One report, which first introduced the CHEMS, provides hazard scores and hazard priorities for 158 chemical substances [22]. Out of these 158 substances, we excluded chemicals that did not have a CAS number and did not have a risk phrase, which was needed to evaluate the EURAM hazard score. After excluding 18 chemicals, we compared the results of each CRS method using 140 chemical substances.

### 2.7. Statistical Analysis

We used a Pearson correlation analysis to perform the correlation analysis between the variables used in the MD calculation and for the comparison of chemical hazard scores between the CRS methods. The comparisons between chemical classes, which were divided into four classes using classification methods, were conducted using the Kappa statistic. The calculation of MD was performed using Minitab version 18.0 (Minitab Inc., Pennsylvania, USA), and other statistical analyses were conducted using SPSS version 25.0 (IBM, New York, USA).

## 3. Results

Table 4 shows the results of the correlation analysis between the MDs of 3028 chemicals and the 10 quantified health hazard variables. The highest correlation variables were skin corrosion/irritation and serious eye damage/eye irritation, with r = 0.701 (*p* < 0.001). There were also strong correlations between acute toxicity (oral) and acute toxicity (dermal) (r = 0.597, *p* < 0.001) and between specific target organ toxicity (single exposure) and specific target organ toxicity (repeated exposure) (r = 0.529, *p* < 0.001). As a result of the correlation between the MD and each variable, the correlation coefficient represented the range of 0.262 to 0.524, and all coefficients were statistically significant.

Table 5 presents the top 10 chemicals that had large MDs. The chemical that had the largest MD was bis(2-chloroethyl) sulfide (MD = 407.69), and bis(2-chloroethyl) methylamine (MD = 264.88) and aziridine (MD = 262.26) followed. Most of the high-ranking chemicals had Category 1 and Category 2 rankings. In particular, it was found that bis(2-chloroethyl) sulfide, with the largest MD, had Category 1 rankings for all variables except acute toxicity (oral).

**Figure 1:**
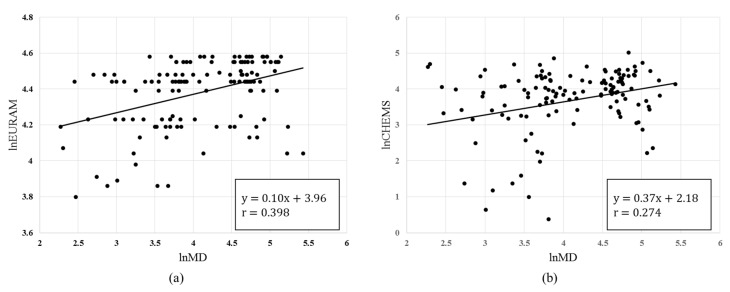
The results of the correlation analysis between the hazard scores calculated using the chemical ranking and scoring methods: (**a**) comparison between Mahalanobis distance (MD) and European Union Risk Ranking Method (EURAM) and (**b**) comparison between MD and Chemical Hazard Evaluation for management Strategies (CHEMS).

Figure 1 shows the results of the correlation analysis between the hazard scores calculated using the MD, EURAM, and CHEMS. The Pearson correlation coefficients for the MD and EURAM and for the MD and CHEMS were 0.445 (*p* < 0.001) and 0.339 (*p* < 0.001), respectively. Both coefficients were statistically significant, but were not large enough to indicate that there was a strong correlation.

Table 6 shows the results of the intermethod agreement analysis between chemical classes, which were divided into four classes using classification methods. The range in the quadratic weighted Kappa statistic was 0.061 to 0.441 in the comparison between the MD and EURAM, and was 0.221 to 0.263 in the comparison between the MD and CHEMS. The Kappa statistic tended to increase as the degree of weight increased, but the Kappa statistics did not reach high enough values to say that there was high agreement.

Table 7 shows the arithmetic mean and 95% confidence interval (CI) of the hazard scores by types of chemicals classified according to the Korea Chemicals Control Act. For the MD, the mean score of chemicals requiring preparation for accidents was the highest at 113.53 (95% CI: 95.49, 131.58), and it was about 1.6 times higher than the average of all chemicals and 2.4 times higher than the average of other chemicals. The hazard scores using the EURAM showed that the score of restricted chemicals was the highest at 95.88 (95% CI: 93.74, 98.02), and the mean scores of chemicals managed by the Act were higher than the average of the total substances and other chemicals. For the CHEMS, the mean score of prohibited chemicals was the highest at 84.08 (95% CI: 43.00, 125.16), and the average of chemicals requiring preparation for accidents was lower than the average score of the total chemicals.

Table 8 presents the proportion of chemicals selected as highly hazardous in each method that were included in the chemical list of the Korea Chemicals Control Act. In the case of the MD, 42 of 66 chemicals (63.6%) managed by the Act were classified as Grade 3 or Grade 4. However, for the EURAM and CHEMS, 26 (39.4%) and 13 chemicals (19.7%) were classified as Grade 3 or Grade 4, respectively.

## 4. Discussion

This study presented a new method to score the health hazard ranking of chemicals using the MTS and the information from the GHS, and compared the results with the existing methods. The MD had a statistically significant positive correlation with all 10 types of health hazard variables, and chemicals with higher health hazard categories showed larger MDs. The scores of chemicals using the MD showed low correlation coefficients with the scores of the EURAM and CHEMS, with r = 0.398 and r = 0.274, respectively. However, the proportion of chemicals managed by the Korea Chemicals Control Act that were given a high rating appeared to be highest when the MD was used.

A correlation analysis of the MD and 10 types of health hazard variables was conducted to confirm that the MD included each variable adequately. The correlation coefficient for the final score and each variable ranged from 0.580 to 0.911 in previous studies, such as that by Shin et al. [23], and ranged from 0.32 to 0.76 in a previous study by Swanson et al. [4]. In this study, the correlation coefficients for the MD and the 10 health hazard variables ranged from 0.262 to 0.524, and they were all statistically significant. Since the MD had a significantly positive correlation with all variables, we deduced that the MD properly included the information about all variables. Furthermore, since no variable had an especially high correlation coefficient, it could be expected that the ranking results will not be changed by some variables.

Comparing the agreement of the total score of chemicals between the MD and the EURAM and CHEMS, the correlation coefficients showed low correlation with values of 0.398 and 0.274, respectively (Figure 1). After classification into four chemical grades, the highest Kappa statistics between the MD and the EURAM and CHEMS were 0.441 and 0.263, respectively (Table 6). This appeared to be because the MD only uses the health hazard information of chemicals, while the EURAM and CHEMS use health hazard information and exposure possibility when calculating the hazard scores of chemicals [4,12]. In addition, the CHEMS had a larger difference with the results of the MD because it additionally uses environmental hazard information. Generally, in CRS methods considering health hazards and environmental hazards together, carcinogenic heavy metals that have high accumulation tendencies are typically ranked higher [22,24,25,26]. However, when the purpose is to evaluate health hazards, such as calculating the hazard ranking of chemicals used at workplaces, including the environmental hazard as a priority-ranking factor is unsuitable. Therefore, information that needs to be considered varies depending on the purpose of the management of chemicals, and selectively using a method as needed seems feasible.

The Korea Chemicals Control Act divides highly harmful chemicals into the following four categories: chemicals requiring preparation for accidents (CRPA); toxic chemicals (TC); prohibited chemicals (PC); restricted chemicals (RC). Because these chemicals are highly harmful, it can be expected that the MD, EURAM score, and CHEMS score of these chemicals will be higher than the other chemicals which are not managed by the Act. In Table 7, the MD and EURAM scores of the chemicals managed by law were higher than the mean of the total and other chemicals. However, in the case of CHEMS, the mean score of CRPA was less than the total mean, and the mean score of other chemicals was not significantly lower than the mean of CRPA. Also, in Table 8, the true positives of MD were about 1.5 times more frequent than those of the EURAM. Based on the above results, therefore, we can say that the MD more accurately reflects the hazardous nature of chemicals than the EURAM and CHEMS.

The method of using the MD has several strengths. First, the MD can comprehensively consider diverse variables related to health hazards. The EURAM only applies one risk phrase value with the highest health hazard when calculating the health effect score. Therefore, as other information, except the most hazardous information, is not considered in the scoring, the actual hazard of the chemicals could be underestimated in the case of chemicals with various health effects. For example, terbufos (CAS No. 13071-79-9) was classified as Category 2 for reproductive toxicity and was classified as Category 1 for acute toxicity (oral) and acute toxicity (dermal). When evaluating terbufos using the EURAM, it was calculated as ranking 40th among 140 substances, because only the information regarding reproductive toxicity was considered. However, when terbufos was evaluated using the MD, it was calculated as 21st, which is a higher ranking than that calculated by the EURAM, because information about both reproductive toxicity and acute toxicity was considered. The second strength is that the calculated result takes the correlation between variables into account. According to the correlation analysis in this study (Table 4), the correlation coefficient for two variables, namely acute toxicity (oral) and acute toxicity (inhalation), was 0.284 (*p* < 0.001). In other words, these two variables shared about 28% of the characteristics. However, the CHEMS may overestimate the actual hazard of chemicals because it independently considers information regarding acute toxicity (oral) and acute toxicity (inhalation) for the ranking calculation. For example, the acute toxicity (oral) and acute toxicity (inhalation) of formaldehyde (CAS No. 50-00-0) were classified as Category 4 and Category 2, respectively. When evaluating formaldehyde using the CHEMS, it was calculated to be ranked 7th among 140 substances, because these two variables were applied independently. However, since the correlation between the two variables was considered when evaluating formaldehyde using the MD, the chemical was ranked as 16th, which is a lower ranking than that given by the CHEMS. Consequentially, the possibility of obtaining a more accurate priority ranking of chemicals increases by using the MD rather than the existing methods.

The MD also has some limitations. Even if the same variables are used in the evaluation, the MD of each chemical can differ according to the method used for quantifying the health hazard categories. In particular, the health hazard ranking of chemicals can differ significantly according to the quantification of Classification Not Possible. Yoko Kubota et al. found that if Classification Not Possible is given a score corresponding to the level of Category 2, then the effect of Classification Not Possible may be overestimated. If Classification Not Possible is allocated a value near that of Category 3, then it will mostly be appropriate [27,28]. Therefore, this study quantified Classification Not Possible as 30, which is similar to the value of Category 3, to obtain appropriate results. Other than this, the ranking results of chemicals can be affected because the MD differs according to the quantifying method of each category; thus, it appears that additional studies about this are needed.

## 5. Conclusions

In this study, we presented a new method using the MTS to overcome the limitations of existing CRS methods. The MTS method produced chemical rankings that took into accounts the correlation between variables related to chemical health hazards, and the MDs were shown to reflect each variable well. The agreement with existing methods, such as the EURAM and CHEMS, was not high, but the proportion of chemicals managed by the Korea Chemicals Control Act that were given a high rating appeared to be highest when the MD was used. These results indicated that the new method evaluates the health hazards of chemicals more accurately, and we expect that the MTS method could be applied to a greater range of chemicals than the existing methods.

## Figures and Tables

**Table 1:** Types of health hazard variables according to the Globally Harmonized System.

No.	Variables	Categories							
1	Acute toxicity (oral)	1	2	3	4	5	Not Classified	Classification Not Possible	Not Applicable
2	Acute toxicity (dermal)	1	2	3	4	5			
3	Acute toxicity (inhalation)	1	2	3	4	5			
4	Skin corrosion/irritation	1	2	-	-	-			
5	Serious eye damage/eye irritation	1	2	-	-	-			
6	Germ cell mutagenicity	1A	1B	2	-	-			
7	Carcinogenicity	1A	1B	2	-	-			
8	Reproductive toxicity	1A	1B	2	-	-			
9	Specific target organ toxicity (single exposure)	1	2	3	-	-			
10	Specific target organ toxicity (repeated exposure)	1	2	-	-	-			

**Table 2:** The quantification results of health hazard variables.

Categories	Category 1	Category 2	Category 3	Category 4	Category 5	Not Classified	Classification Not Possible	Not Applicable
Score	100	100/2 = 50	100/3 = 33.3	100/4 = 25	100/5 = 20	10	30	1

**Table 3:** The process of selecting chemicals that are relatively less hazardous.

Criteria	Value
Number of ‘Category 1’ rankings	0
Number of ‘Category 2’ rankings	0
Total score of 10 variables related to health hazards	<280

**Table 4:** The results of the correlation analysis between the Mahalanobis distances (MDs) of 3028 chemicals and the 10 quantified health hazard variables.

Variables	1	2	3	4	5	6	7	8	9	10	11
1. MD	1										
2. Acute toxicity (oral)	0.262	1									
3. Acute toxicity (dermal)	0.294	0.597	1								
4. Acute toxicity (inhalation)	0.307	0.284	0.248	1							
5. Skin corrosion/irritation	0.497	0.103	0.149	0.194	1						
6. Serious eye damage/eye irritation	0.487	0.045	0.039	0.197	0.701	1					
7. Germ cell mutagenicity	0.303	0.106	0.123	0.042	0.076	0.039	1				
8. Carcinogenicity	0.444	0.048	0.068	0.000	0.364	−0.015	0.371	1			
9. Reproductive toxicity	0.524	0.045	0.056	−0.017	−0.022	−0.008	0.252	0.320	1		
10. Specific target organ toxicity (single exposure)	0.457	0.222	0.128	0.267	0.129	0.201	0.095	0.155	0.213	1	

**Table 5:** Top 10 chemicals with large Mahalanobis distances (MDs).

Chemical Name	MD	Acute Toxicity (Oral)	Acute Toxicity (Dermal)	Acute Toxicity (Inhalation)	Skin Corrosion/Irritation	Serious Eye Damage/Eye Irritation	Germ Cell Mutagenicity	Carcinogenicity	Reproductive Toxicity	Specific Target Organ Toxicity (Single Exposure)	Specific Target Organ Toxicity (Repeated Exposure)
Bis(2-chloroethyl)sulfide	407.69	Category 2	Category 1	Category 1	Category 1	Category 1	Category 1	Category 1	Category 1	Category 2	Category 1
Bis(2-chloroethyl)methylamine	264.88	Category 2	Category 1	Category 1	Category 1	Category 1	Category 1	Category 1	Classification Not Possible	Category 1	Classification Not Possible
Aziridine	262.26	Category 2	Category 1	Category 1	Category 1	Category 1	Category 1	Category 2	Category 2	Category 1	Category 1
Sodium dichromate	253.33	Category 3	Category 3	Category 2	Category 1	Category 1	Category 2	Category 1	Not Classified	Category 1	Category 1
Diethyl sulfate	251.99	Category 4	Category 3	Classification Not Possible	Category 1	Category 1	Category 1	Category 1	Classification Not Possible	Category 2	Classification Not Possible
Chloromethylmethyl ether	233.65	Category 4	Classification Not Possible	Category 1	Category 1	Category 1	Category 2	Category 1	Classification Not Possible	Category 1	Classification Not Possible
Phenol	227.53	Category 4	Category 3	Classification Not Possible	Category 1	Category 1	Category 1	Not Classified	Category 1	Category 1	Category 1
Captafol	221.19	Category 5	Not Classified	Classification Not Possible	Category 2	Category 2	Category 1	Category 1	Not Classified	Classification Not Possible	Category 1
1,4-dichloro-2-butene	214.42	Category 3	Category 3	Category 1	Category 1	Category 1	Category 2	Category 1	Classification Not Possible	Category 1	Category 1
dieldrin	213.48	Category 1	Category 1	Category 1	Classification Not Possible	Classification Not Possible	Not Classified	Not Classified	Category 1	Category 1	Category 1

**Table 6:** Kappa statistics according to the classification methods and the weighted methods.

Weighting	MD ^1^ vs. EURAM ^2^				MD ^1^ vs. CHEMS ^3^			
	Equal Interval	Quintile	Jenks Natural Breaks	Geometrical Interval	Equal Interval	Quintile	Jenks Natural Breaks	Geometrical Interval
Unweighted	−0.045	0.200	0.159	0.184	0.224	0.171	0.204	0.203
Linear weighted	0.010	0.293	0.284	0.319	0.217	0.223	0.224	0.247
Quadratic weighted	0.061	0.380	0.392	0.441	0.221	0.263	0.225	0.260

^1^ MD: Mahalanobis distance. ^2^ EURAM: European Union Risk Ranking Method. ^3^ CHEMS: Chemical Hazard Evaluation for Management Strategies.

**Table 7:** Arithmetic mean and 95% confidence interval (CI) of hazard scores by types of chemicals classified according to the Korea Chemicals Control Act.

Chemical Types	N	MD ^1^		EURAM ^2^		CHEMS ^3^	
		Mean	95% CI	Mean	95% CI	Mean	95% CI
Total	140	72.10	63.99, 80.21	79.57	76.71, 82.44	51.50	46.60, 56.39
CRPA ^4^	26	113.53	95.49, 131.58	85.71	80.85, 90.57	49.27	38.62, 59.92
TC ^5^	60	99.82	87.66, 111.97	84.72	80.86, 88.59	59.93	52.75, 67.12
PC ^6^	3	88.43	48.37, 128.50	85.16	44.45, 125.87	84.08	43.00, 125.16
RC ^7^	5	87.03	20.26, 153.79	95.88	93.74, 98.02	66.91	39.94, 93.89
OC ^8^	74	48.19	39.65, 56.73	75.15	70.95, 79.35	45.10	38.37, 51.83

^1^ MD: Mahalanobis distance. ^2^ EURAM: European Union Risk Ranking Method. ^3^ CHEMS: Chemical Hazard Evaluation for Management Strategies. ^4^ CRPA: chemicals requiring preparation for accidents. ^5^ TC: toxic chemicals. ^6^ PC: prohibited chemicals. ^7^ RC: restricted chemicals. ^8^ OC: other chemicals.

**Table 8:** The proportion of chemicals selected as highly hazardous in each method that were included in the chemical list of the Korea Chemicals Control Act.

Regulation	Indicators	Chemical Ranking and Scoring Methods		
		MD ^1^	EURAM ^2^	CHEMS ^3^
Korea Chemicals Control Act	True positive ^4^	63.6% (42/66)	39.4% (26/66)	19.7% (13/66)
	False positive ^5^	13.5% (10/74)	12.2% (9/74)	17.6% (13/74)

^1^ MD: Mahalanobis distance, ^2^ EURAM: European Union Risk Ranking Method, ^3^ CHEMS: Chemical Hazard Evaluation for Management Strategies, ^4^ True positive = $\frac{\mathrm{number}\;\mathrm{of}\;\mathrm{chemicals}\;\mathrm{classified}\;\mathrm{as}\;\mathrm{Grade}\;3\;\mathrm{or}\;\mathrm{Grade}\;4\;\mathrm{in}\;\mathrm{each}\;\mathrm{method}}{\mathrm{number}\;\mathrm{of}\;\mathrm{chemicals}\;\mathrm{controlled}\;\mathrm{by}\;\mathrm{the}\;\mathrm{act}}$, ^5^ False positive = $\frac{\mathrm{number}\;\mathrm{of}\;\mathrm{chemicals}\;\mathrm{classified}\;\mathrm{as}\;\mathrm{Grade}\;3\;\mathrm{or}\;\mathrm{Grade}\;4\;\mathrm{in}\;\mathrm{each}\;\mathrm{method}}{\mathrm{number}\;\mathrm{of}\;\mathrm{chemicals}\;\mathrm{do}\;\mathrm{not}\;\mathrm{controlled}\;\mathrm{by}\;\mathrm{the}\;\mathrm{act}}.$
